# Genital Inflammatory Responses in Women Living with HIV Randomized to Copper or Levonorgestrel Intrauterine Contraceptives: A secondary analysis of a randomized trial

**DOI:** 10.64898/2026.05.24.26353969

**Published:** 2026-05-26

**Authors:** Anna-Ursula Happel, Jo-Ann S. Passmore, Musalula Sinkala, Shameem Z. Jaumdally, Hoyam Gamieldien, Nai-Chung Hu, Nontokozo Langwenya, Heidi E. Jones, Donald R. Hoover, Landon Myer, Catherine S. Todd

## Abstract

**Background:**

Intrauterine contraceptives (IUCs) are effective, but effects on genital inflammation among women living with HIV (WLHIV) by antiretroviral therapy (ART) use are unclear. We evaluated the longitudinal effects of copper IUC (CLIUC) and the levonorgestrel intrauterine system (LNGLIUS) on cervicovaginal cytokine profiles in a secondary analysis of a randomized trial ( NCT01721798 , 2013–2016).

**Methods:**

Cervicovaginal secretions were collected from 100 WLHIV (nonLART usersL=L48; ART usersL=L52) randomized 1:1 to ClZIUC or LNGlZIUS. TwentyLeight cytokines were measured prior to insertion and 3- and 6-months postLinsertion. Cytokine concentrations at each followLup visit were compared with baseline, using participant fixedLeffects models stratified by ART status.

**Results:**

At enrollment, nonLART users had higher average concentrations of most cytokines (21/28) than women using ART. Among non-ART users, IUC use was not associated with cytokine increases; only MCPL1 increased significantly at 3 months among CLIUC users (log _10_ geometric mean ratio 0.77, 95%CI 0.38–1.17), while none increased with LNG-IUS use. Among ART users, C-IUC insertion resulted in broad and sustained cytokine increases at both 3 (16/28) and 6 months (15/28). At month 3, the largest increases in log _10_ geometric mean were observed for IL-6 (1.04, 0.72-1.36), RANTES (0.97, 0.54-1.40), MCP-1 (0.71, 0.46-0.96), MIP-1α (0.66, 0.37-0.94), and G-CSF (0.63, 0.36-0.89), which was maintained until month 6. Cytokine changes following LNGLIUS insertion were minimal (IL-5, month 3).

**Conclusions:**

Among ART users, CLIUC is associated with increases in cervicovaginal cytokines, across functional classes. This supports LNGLIUS as a less inflammatory option for WLHIV to minimize genital immune activation.

## Full Text Availability

The license terms selected by the author(s) for this preprint version do not permit archiving in PMC. The full text is available from the preprint server.

